# Acute kidney injury induced by thrombotic microangiopathy in a patient with hemophagocytic lymphohistiocytosis

**DOI:** 10.1186/s12882-015-0217-z

**Published:** 2016-01-06

**Authors:** Myoung Nam Bae, Dae Hun Kwak, Se Jun Park, Bum Soon Choi, Cheol Whee Park, Yeong Jin Choi, Jong Wook Lee, Chul Woo Yang, Yong-Soo Kim, Byung Ha Chung

**Affiliations:** Division of Nephrology, Department of Internal Medicine, College of Medicine, Seoul St. Mary’s Hospital, The Catholic University of Korea, 222 Banpodae-ro, Seocho-gu, Seoul 137-040 Korea; Department of Hospital Pathology, College of Medicine, Seoul St. Mary’s Hospital, The Catholic University of Korea, 222 Banpodae-ro, Seocho-gu, Seoul 137-040 Korea; Division of Hematology, Department of Internal Medicine, College of Medicine, Seoul St. Mary’s Hospital, The Catholic University of Korea, 222 Banpodae-ro, Seocho-gu, Seoul 137-040 Korea; Department of Internal Medicine, College of Medicine, Seoul St. Mary’s Hospital, The Catholic University of Korea, 222 Banpodae-ro, Seocho-gu, Seoul 137-040 Korea

**Keywords:** Lymphohistiocytosis, Hemophagocytic, Thrombotic microangiopathy, Acute kidney injury

## Abstract

**Background:**

Hemophagocytic lymphohistiocytosis (HLH) is a fatal clinical syndrome characterized by excessive immune activation and inflammation. It is frequently complicated by acute kidney injury (AKI) that often develops as acute tubular necrosis (ATN). Meanwhile, renal thrombotic microangiopathy (TMA) is a rare pathologic finding that mostly occurs in hemolytic uremic syndrome or thrombotic thrombocytopenic purpura. There are only few reports on TMA developing in patients with HLH. We present here a rare case of TMA associated HLH.

**Case presentation:**

A 60-year-old woman was admitted for a fever of unknown origin that had persisted for several weeks. She presented with AKI and pancytopenia. Clinical, laboratory and bone marrow biopsy findings met the criteria of HLH. Kidney biopsy showed TMA and minimal ATN, which suggested that the primary cause of AKI was TMA in this case. Because of sustained oliguria, we initiated hemodialysis (HD) and also decided to use chemotherapy composed of dexamethasone, etoposide and cyclosporine for treatment of HLH. Six months after the initiation of chemotherapy, pancytopenia was completely resolved, indicating the resolution of HLH. At the same time, serum creatinine decreased to a normal range without the need for HD, suggesting the resolution of TMA.

**Conclusion:**

We report a case of renal TMA associated HLH. This case suggests that renal TMA should be considered as a primary cause of AKI in patients with underlying HLH.

## Background

Hemophagocytic lymphohistiocytosis (HLH) is a very rare clinical syndrome characterized by excessive immune activation and inflammation [1–3]. Severe HLH is commonly accompanied by various organ dysfunctions including manifestation of acute kidney injury (AKI). Indeed, the prevalence of AKI in severe HLH has been reported as 30 ~ 50 %, and acute tubular necrosis (ATN) by sepsis or dehydration due to fever has been suspected as the renal pathology in HLH with AKI [1, 2, 4]. Meanwhile, thrombotic microangiopathy (TMA) is rarely detected in the renal pathology state, which develops secondary to hematologic, autoimmune or infectious disease. Hemolytic uremic syndrome (HUS) or thrombotic thrombocytopenic purpura (TTP) is a well characterized disorder associated with TMA [5, 6]. However, TMA is rarely detected in patients with HLH, despite the frequent development of renal complications.

In the present case, we encountered AKI developed as TMA in a patient diagnosed with HLH. We successfully treated both the TMA and HLH using cytotoxic therapy including dexamethasone, etoposide and cyclosporine.

## Case presentation

A 60-year-old woman was admitted because of persistent fever of unknown origin lasting for several days. She had been in a good state of health prior to admission and had no specific underlying disease. She had no related family history and medication history. Upon physical examination, the vital signs were as follows: blood pressure, 120/80 mmHg; pulse rate, 87 beats per minute; and body temperature, 37.8 °C. There were no palpable lymph nodes in the neck, armpit and groin areas. Both legs had pitting edema and petechial rashes. She also complained of diffuse abdominal pain and oliguria that had been present for several days.

**Fig. 1:**
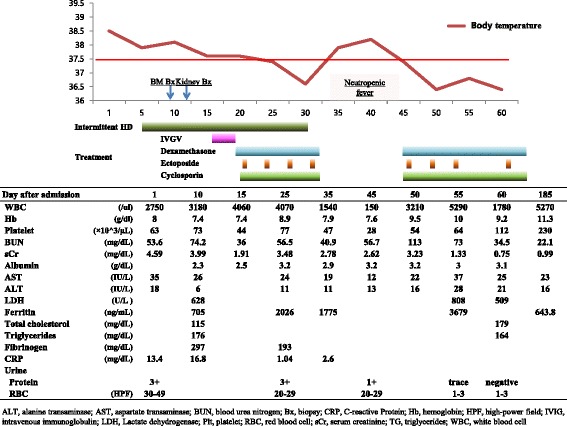
Clinical course and laboratory findings of the patient

Laboratory findings are summarized in Fig. 1. Pancytopenia (white blood cell count, 2750/μL; hemoglobin, 8 g/dL; platelet count, 63,000 /μL) was detected and serum creatinine level was significantly elevated (4.59 mg/dL). Serum ferritin, C-reactive protein and lactate dehydrogenase were increased as well. Prothrombin times (PT), activated partial thromboplastin times (aPTT) and fibrinogen were within normal limits. There was no dyslipidemia, including hypertriglyceridemia. The patient’s soluble interleukin-2 receptor level in serum was 5.2 % (normal range 5–30 %) and natural killer (NK) cells activity was decreased (NK cells activity, 3.7 %; normal range 6–29 %). Urinary protein/creatinine ratio was 906.4 mg/g. Peripheral blood smear showed normocytic normochromic anemia and thrombocytopenia. The examination revealed no schistocytes and anisocytosis, which suggested absence of microangiopathic hemolytic anemia (MAHA). Abdominal computerized tomography (CT) scan for the evaluation of abdominal pain showed no abnormal findings for the bowel, pancreas or the biliary tract. Both kidney sizes were normal without chronic change, but there was significant splenomegaly, multifocal ascites and pleural effusion were detected.

At first, we used broad-spectrum antibiotics for neutropenic fever and initiated hemodialysis for acute renal failure. However, we did not find any etiology for neutropenic fever from urine or blood culture studies. In addition, virus studies, including epstein-barr virus, parvovirus B19, adenovirus, human immunodeficiency virus, hepatitis B and influenza virus were negative on serologic tests. Anti-hepatitis C virus antibody was positive but alanine transaminase and aspartate transaminase levels were normal, which suggested that hepatitis C virus was in an inactive state. The patient showed no evidence of tuberculosis in chest X-ray, sputum acid-fast bacillus (AFB) stain and AFB culture, sputum and urine tuberculosis polymerase chain reaction. Autoimmune disorders such as systemic lupus erythematosus or rheumatoid arthritis were carefully ruled out by clinical symptoms and signs and by autoantibody tests.

**Fig. 2:**
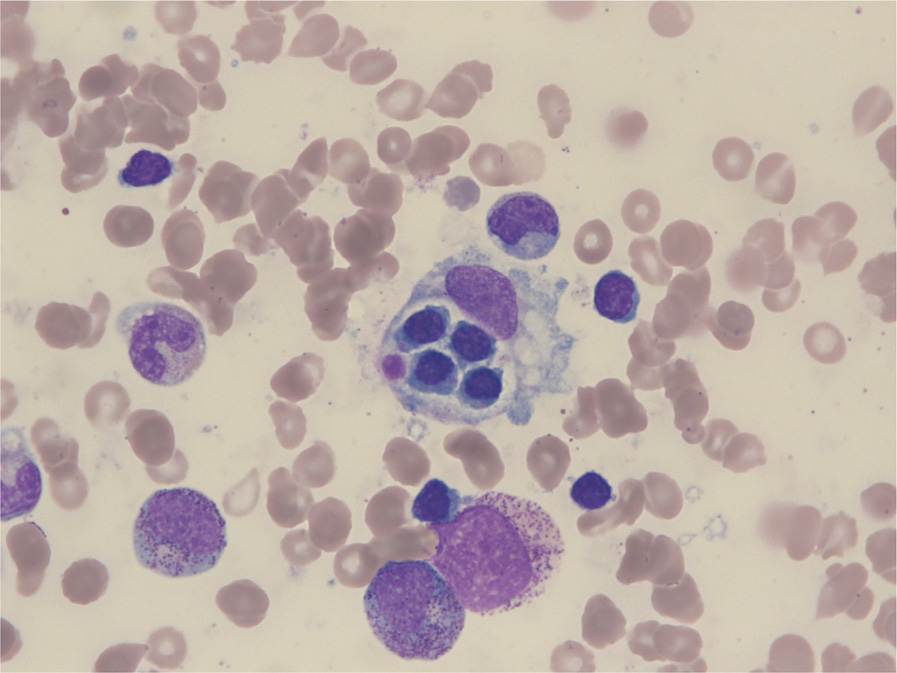
Bone marrow biopsy findings. Microscopic findings of bone marrow aspiration shows hemophagocytosis of red blood cells (Wright-Giemsa stain, ×400)

Consequently, we decided to perform a bone marrow (BM) biopsy to determine the reason for fever and pancytopenia. BM biopsy revealed about 10 % cellularity and numerous histiocytes with engulfed lymphocytes, polymorphonuclear and red blood cells, which were suggestive of hemophagocytosis (Fig. 2). Finally, the patient was diagnosed with HLH based on fever, progressive pancytopenia, hyperferritinemia, splenomegaly, decreased NK cell activity and hemophagocytosis in BM and negative results on viral and autoimmune marker studies.

**Fig. 3:**
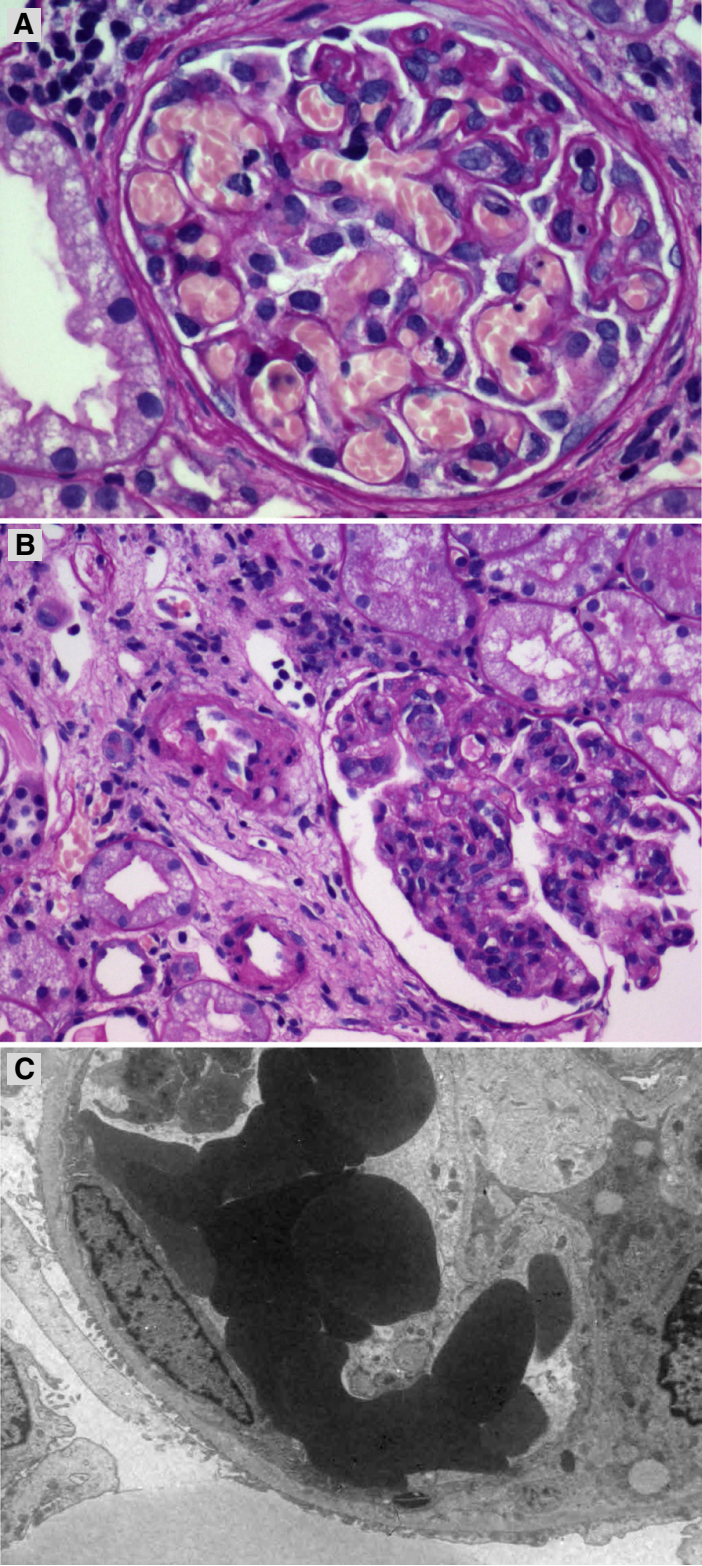
Kidney biopsy findings. **a** Light microscopic findings show capillary lumens filled with fragmented red blood cells and platelet aggregates (H&E stain, ×400). **b** Light microscopic findings show tubules reveal focal moderate atrophy and loss with interstitial fibrosis (H&E stain, ×200). **c** Electron microscopy findings show focal effacement of podocyte foot processes. There are no electron-dense deposits (Original magnification, ×5000)

Meanwhile, a kidney biopsy was performed to investigate the cause of acute kidney injury because renal function did not improve. PT times (PT INR, 1.13) and aPTT times (aPTT, 23.3 seconds) were within normal limits. But complete blood count showed thrombocytopenia. We used prophylactic transfusion of platelets in preparation for a kidney biopsy that could cause bleeding. Under light microscopic examination, glomeruli were slightly enlarged with hypercellularity involving mesangial and endothelial cells. Capillary lumens were frequently filled with fragmented red blood cells and platelet aggregates (Fig. 3a). Tubules revealed focal moderate atrophy and loss with interstitial fibrosis (Fig. 3b). In an immunofluorescence study, staining for immunoglobulin G, immunoglobulin M, immunoglobulin A, C4, fibrinogen, kappa and lambda were negative. In electron microscopy, no electron-dense deposits were found and epithelial foot process showed focal marked effacement (Fig. 3c). The above histologic findings were compatible with renal TMA.

At this time, we planned 6 cycles of cytotoxic therapy comprised of dexamethasone, etoposide at 100 mg/m^2^ and cyclosporine at 200 mg/day for the treatment of HLH based on the HLH-2004 protocol [7]. Dexamethasone started at 10 mg/ m^2^ for 2 weeks, and then reduced to 50 % of initial dose every 2 weeks. As etoposide is cleared by both renal and hepatic routes, dose adjustment of etoposide based on renal function is recommended for the HLH-2004 protocol. We started at etoposide 100 mg/m^2^ with dose reductions of 25 % based on the patient’s renal function. The general condition of the patient gradually improved and the fever subsided 7 days after the initiation of cytotoxic therapy. Concomitantly, urine output also increased and we ceased hemodialysis at 28 days from the first initiation of hemodialysis. After the 4th cycle of etoposide treatment, she suffered neutropenic fever due to vancomycin-resistant enterococci infection, which was successfully treated with linezolid and recombinant human granulocyte colony stimulating factor. After 7 cycles of etoposide treatment, she was discharged with normal kidney function and no signs of fever or neutropenia. At 6 months after the initial presentation, she showed no signs of HLH recurrence. A bone marrow aspiration and biopsy was done and the bone marrow biopsy looked normal cellular with normal hematopoiesis. Her serum creatinine level were in the normal range (0.99 mg/dL).

## Discussion

In this report, we describe a patient admitted for persistent fever, edema and abdominal discomfort. She also complained of oliguria for the past several days. Laboratory findings showed pancytopenia combined with AKI. The cause of the neutropenic fever was diagnosed as HLH and the cause of the acute renal failure was renal TMA. Cytotoxic therapy targeting HLH improved both neutropenic fever and AKI, suggesting that TMA may have developed secondary to HLH.

With regard to the neutropenic fever, we concluded that a severe bacterial infection might have been the main cause. Therefore, we also suspected that AKI might have developed, as associated with the infection and volume depletion due to fever and poor oral intake over the past few days. Contrast dye used for abdominal CT could have contributed to the aggravation of acute kidney injury. However, we could not find any evidence of infection in the blood, urine culture study, and radiologic exam. In addition, broad spectrum antibiotics did not improve pancytopenia even though the fever partially subsided. In addition, renal function did not improve with fluid therapy and sustained hemodialysis. As a result, we decided to perform kidney biopsy for acute kidney injury of unknown origin and bone marrow biopsy for fever of unknown origin as well. Kidney biopsy showed pathologic findings compatible with TMA. ATN, which is the main finding in cases of infection associated with AKI or pre-renal AKI, was minimal. This suggested that the primary cause of AKI in this case was not pre-renal or infection-associated AKI but TMA. Interestingly, bone marrow biopsy revealed active hemophagocytosis. From the physical exam, radiologic findings and laboratory findings such as fever, progressive pancytopenia, hyperferritinemia, and splenomegaly, we diagnosed HLH in this patient.

Renal TMA frequently manifests as AKI in nearly 90 % of the cases [8]. This disease entity describes a pathological process of microvascular thrombosis due to endothelial injury. Usually, it results from HUS, TTP or malignant hypertension, all of which may induce significant endothelial injury in microvasculature [5, 6, 8]. Her laboratory findings showed that MAHA was negative and the stool study for *E.coli* O157:H7 as well as the streptococcus pneumonia urinary antigen test were negative. The patient’s blood pressure was normal and clotting parameters were within normal limits as well. Therefore, we could rule out HUS, atypical HUS, TTP, disseminated intravascular coagulation and malignant hypertension for the etiology of renal TMA.

Our next consideration was whether the development of TMA was related to underlying HLH. Acute kidney injury is frequent in severe HLH cases and results from inflammatory or ischemic lesions of the renal tubules [2, 9]. The most frequent presentation of renal damage in HLH is ATN [2, 4, 10]. Some authors have described intrarenal lesions associated with hemophagocytosis [11]. Nephrotic syndrome can also occur but seems to be less common than ATN and collapsing glomerulopathy, minimal-change disease and focal segmental glomerulosclerosis with marked podocytosis have been reported [2, 4, 12]. Vascular lesions have been also reported and in a few reports it was a feature of TMA [4, 13].

Clinical features in renal TMA in HLH patients are summarized in Table 1. AKI was present in 5/5 cases and oliguria in 2/5 cases. Proteinuria and microscopic hematuria was present in 5/5 and 4/5 cases, respectively. Our case showed generalized edema, proteinuria and hematuria compatible with nephritic syndrome. Nephritic syndrome associated with renal TMA in HLH is more frequent than nephrotic syndrome. Dialysis therapy was required for two patients. Four patients recovered and death occurred in one case.

**Table 1:** Clinical summary of renal TMA in HLH

Patient number	1	2	3	4	5
Age	63	18	24	18	60
Gender	Female	Female	Male	Female	Female
Race	Caucasian	Asian	NA	Asian	Asian
Etiology of HLH	CMV	Idiopathic	Parvovirus B19	Idiopathic	Idiopathic
Renal manifestation					
Proteinuria	Positive	Positive	Positive	Positive	Positive
24 hr urine	7.3 g/day	9.6 g/day	NA	>3.5 g/day	NA
Urine PCR	NA	NA	510 mg/g	NA	906 mg/g
Microscopic hematuria	Positive	Positive	Negative	Positive	Positive
Oliguria	Absent	NA	Absent	Absent	Yes
Dialysis	HD	No	No	No	HD
Treatment					
Steroids	Yes	Yes	Yes	Yes	Yes
IVIG	Yes	Yes	Yes	Yes	Yes
Cytotoxic agents	No	No	No	No	Yes
Outcome	Dead	Cured	Cured	Cured	Cured
Reference	Thaunat et al.,[4]	Thaunat et al.,[4]	Ardalan et al.,[13]	Chiang et al.,[18]	Our case

*CMV* cytomegalovirus, *HD* hemodialysis, *HLH* Hemophagocytic lymphohistiocytosis, *hr* hour, *IVIG* intravenous immunoglobulin, *NA* not available, *PCR* Protein:Creatinine Ratio

Pathogenesis of HLH could explain the development of TMA. The release of large amounts of tumor necrosis factor (TNF), an important inflammatory cytokine, by activated macrophages was detected during the acute phase of HLH [4, 14]. Renal endothelial cells are activated by TNF and acute endothelial cell changes may lead to altered vascular reactivity, permeability, adherence of leukocytes, coagulation and microvascular vasomotor autoregulation [8, 14, 15]. Therefore, we postulate that these inflammatory processes may cause severe vascular endothelial cell injury and then induce platelet adhesion on its surface for the initial event of platelet aggregates [8, 15]. Nevertheless, TMA in HLH has been rarely reported maybe because diagnostic biopsy has been rarely done in HLH patients. Hence it is possible that TMA in HLH may occur with greater frequency than previously known.

After the diagnosis of HLH, we decided to perform cytotoxic therapy targeting HLH. The therapy was based on the HLH-2004 protocol, which was comprised of a cocktail of etoposide, dexamethasone, and cyclosporine [16, 17]. After the treatment, pancytopenia resistant to broad spectrum antibiotics and conservative therapies improved. In addition, renal function gradually improved, and serum creatinine level decreased to a normal range after 6 cycles of the cytotoxic therapy. As we mentioned above, we thought the cause of TMA was HLH; hence it is possible that the therapies applied in this case effectively controlled cytokine burst and blocked vascular endothelial cell injury, resulting not only in the improvement of symptoms related to HLH, but also in the improvement of AKI associated with renal TMA.

## Conclusions

In this case, we report TMA associated HLH which was successfully treated with cytotoxic therapy targeting HLH. This case suggests that for acute kidney injury in patients with underlying HLH, underlying renal TMA may be considered as the cause of AKI.

### Consent

Written informed consent was obtained from the patient for publication of this case report and any accompanying images. A copy of the written consent is available for review by the Editor of this journal.

## Abbreviations

AFB: Acid-fast bacillus
AKI: Acute kidney injury
aPTT: Activated partial thromboplastin times
ATN: Acute tubular necrosis
BM: Bone marrow
CT: Computerized tomography
HD: Hemodialysis
HLH: Hemophagocytic lymphohistiocytosis
HUS: Hemolytic uremic syndrome
MAHA: Microangiopathic hemolytic anemia
NK: Natural killer
PT: Prothrombin times
TMA: Thrombotic microangiopathy
TNF: Tumor necrosis factor
TTP: Thrombotic thrombocytopenic purpura
